# Advanced age at onset is associated with distinct functional brain reorganization in multiple sclerosis

**DOI:** 10.1093/braincomms/fcag396

**Published:** 2026-10-16

**Authors:** Nicolò Tedone, Paola Valsasina, Alessandro Meani, Paolo Preziosa, Marta Grosselle, Federica Esposito, Massimo Filippi, Maria A Rocca

**Affiliations:** Neuroimaging Research Unit, Institute of Experimental Neuroscience, IRCCS San Raffaele Scientific Institute, 20132 Milan, Italy; Vita-Salute San Raffaele University, 20132 Milan, Italy; Neuroimaging Research Unit, Institute of Experimental Neuroscience, IRCCS San Raffaele Scientific Institute, 20132 Milan, Italy; Neuroimaging Research Unit, Institute of Experimental Neuroscience, IRCCS San Raffaele Scientific Institute, 20132 Milan, Italy; Neuroimaging Research Unit, Institute of Experimental Neuroscience, IRCCS San Raffaele Scientific Institute, 20132 Milan, Italy; Vita-Salute San Raffaele University, 20132 Milan, Italy; Neurology Unit, IRCCS San Raffaele Scientific Institute, 20132 Milan, Italy; Neuroimaging Research Unit, Institute of Experimental Neuroscience, IRCCS San Raffaele Scientific Institute, 20132 Milan, Italy; Neurology Unit, IRCCS San Raffaele Scientific Institute, 20132 Milan, Italy; Neuroimaging Research Unit, Institute of Experimental Neuroscience, IRCCS San Raffaele Scientific Institute, 20132 Milan, Italy; Vita-Salute San Raffaele University, 20132 Milan, Italy; Neurology Unit, IRCCS San Raffaele Scientific Institute, 20132 Milan, Italy; Neurorehabilitation Unit, IRCCS San Raffaele Scientific Institute, 20132 Milan, Italy; Neurophysiology Service, IRCCS San Raffaele Scientific Institute, 20132 Milan, Italy; Neuroimaging Research Unit, Institute of Experimental Neuroscience, IRCCS San Raffaele Scientific Institute, 20132 Milan, Italy; Vita-Salute San Raffaele University, 20132 Milan, Italy; Neurology Unit, IRCCS San Raffaele Scientific Institute, 20132 Milan, Italy

**Keywords:** late-onset multiple sclerosis, adult-onset multiple sclerosis, resting-state functional connectivity, clinical disability, cognitive dysfunction

## Abstract

Functional network reorganization supports compensatory mechanisms in multiple sclerosis, but ageing and later disease onset may alter this balance. Late-onset multiple sclerosis (age at onset ≥50 years) represents a relevant subgroup to examine how disease onset in older age is associated with functional connectivity abnormalities. In this cross-sectional study, we investigated resting-state functional connectivity alterations in late-onset multiple sclerosis versus adult-onset multiple sclerosis and their associations with clinical, cognitive and structural MRI measures. Thirty-four late-onset multiple sclerosis, 169 sex- and disease duration–matched adult-onset multiple sclerosis, and 163 age- and sex-matched healthy controls underwent clinical, neuropsychological (Brief Repeatable Battery of Neuropsychological Tests) and MRI assessment including resting-state functional MRI. Independent component analysis quantified resting-state functional connectivity within 10 large-scale networks. Between-group differences were tested using sex- and scanner-adjusted voxel-wise models (*P* < 0.001, uncorrected, cluster extent ≥10 voxels); resulting abnormal clusters were then examined for exploratory uncorrected associations with Expanded Disability Status Scale, disease duration, cognitive performance, T2-hyperintense lesion volume and normalized brain volume, without correction for multiple comparisons. At MRI acquisition, compared with the adult-onset multiple sclerosis group, the late-onset multiple sclerosis group was significantly older (median age: 60.5 [IQR: 56.6–66.1] versus 35.6 [IQR: 30.0–40.1] years; pFDR = 0.006) and showed higher Expanded Disability Status Scale score (pFDR = 0.016) and higher T2-hyperintense lesion volume (pFDR = 0.028) despite similar disease duration (median: 4.5 [IQR: 1.7–13.2] versus 6.0 [IQR: 2.1–11.8] years; pFDR = 0.894), whereas no significant between-group cognitive difference was found. Both multiple sclerosis groups showed widespread functional connectivity abnormalities versus healthy controls, partly overlapping and partly distinct. Adult-onset multiple sclerosis displayed functional connectivity increases in the executive control and occipital default mode networks. Late-onset multiple sclerosis showed diffuse functional connectivity alterations, including decreases in the left fronto-parietal, thalamic and occipital default mode networks. Exploratory uncorrected correlation analyses included 117 tested associations. In late-onset multiple sclerosis, decreased functional connectivity in the left fronto-parietal network is related to lower normalized brain volume and worse cognition (*P* = 0.02–0.03), whereas altered thalamic network connectivity is related to higher Expanded Disability Status Scale, longer disease duration, and higher T2-hyperintense lesion volume (*P* = 0.01–0.02). These associations did not survive correction for multiple comparisons and should therefore be considered hypothesis-generating. These findings suggest that late-onset multiple sclerosis was associated with a distinct resting-state functional connectivity profile compared with adult-onset multiple sclerosis within a cross-sectional, age-matched healthy control framework, possibly reflecting the combined contribution of multiple sclerosis-related damage, age-related vulnerability and the more severe clinical profile of late-onset multiple sclerosis.

## Graphical Abstract

**Graphical Abstract:**
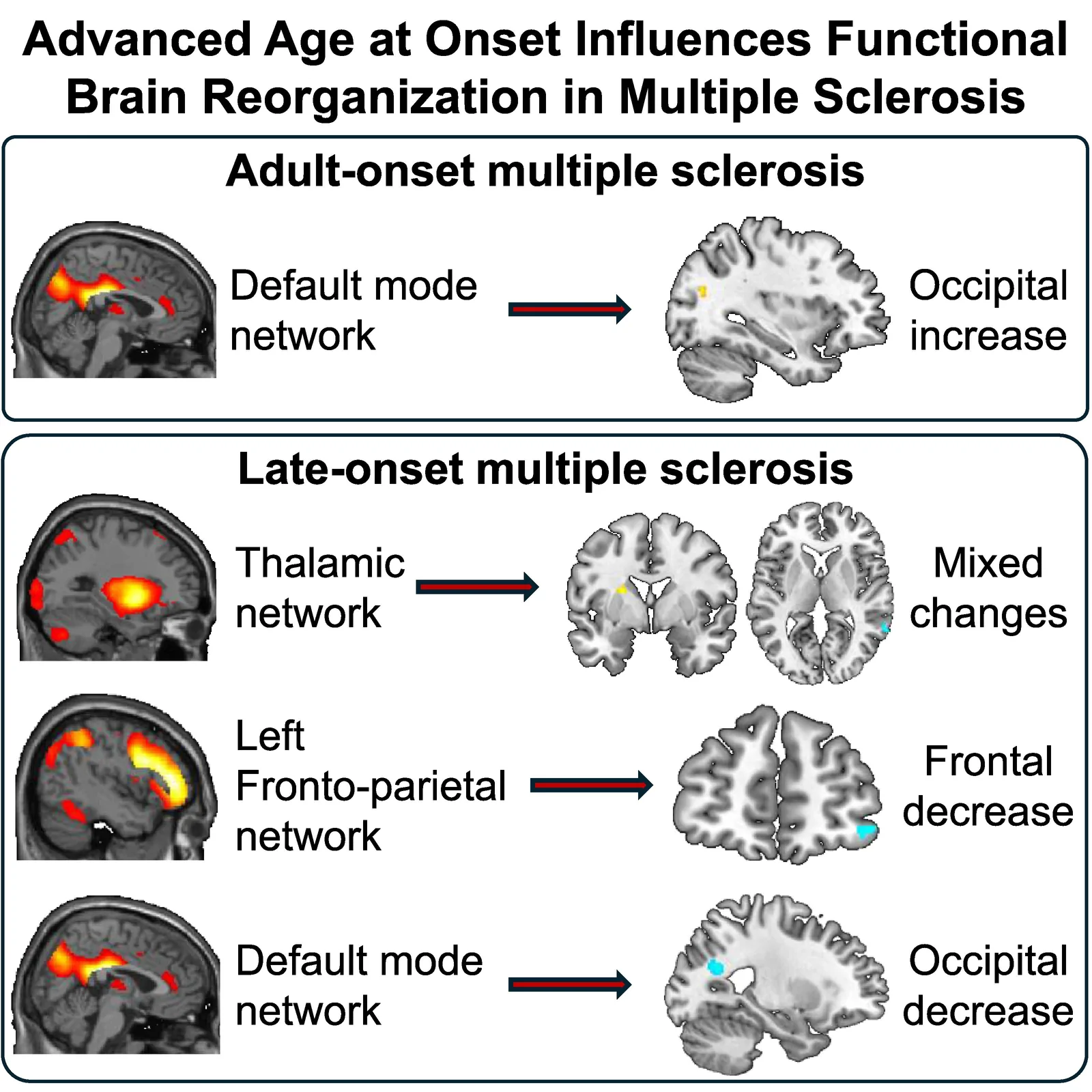

## Teaser

Tedone *et al*. report that late-onset multiple sclerosis (age at onset ≥50 years) is associated with broader network changes than adult-onset disease. These widespread connectivity abnormalities showed exploratory associations with higher disability, worse cognitive performance and greater structural brain damage, suggesting potentially less efficient functional reorganization in late-onset patients.

## Introduction

Functional organization of the brain relies on the coordinated activity of large-scale networks that support motor, sensory and cognitive functions.^1^ Both normal ageing and multiple sclerosis (MS) disrupt this organization, although through distinct but interacting mechanisms.^2-4^ The effects of ageing result in profound modifications in network architecture, including reduced segregation between functional systems, decreased efficiency of long-range communication and compensatory hyperconnectivity in prefrontaland subcortical regions.^2,4^ In multiple sclerosis, network integrity is further challenged by demyelination, axonal loss and neuroinflammation, resulting in altered patterns of activity and connectivity.^3^ Resting-state (RS) functional MRI (fMRI) is one of the principal techniques used to reconstruct functional connectivity (FC) within large-scale brain networks.^5,6^ Accordingly, RS-fMRI has been consistently used in multiple sclerosis to characterize patterns of functional reorganization across disease stages and to examine their relationships with clinical and cognitive outcomes as well as underlying structural damage.^3^

In addition, different ages at onset define distinct multiple sclerosis phenotypes. The typical age of multiple sclerosis onset falls between 20 and 40 years,^7^ a condition referred to as adult-onset multiple sclerosis (AOMS). Around 5% of newly diagnosed patients experience the first clinical manifestations at 50 years of age or later, a condition termed late-onset (LO) multiple sclerosis.^8^ Studies characterizing clinical and cognitive outcomes of late-onset multiple sclerosis (LOMS) reported that these patients generally tend to present with more severe disability, faster progression and more pronounced cognitive deficits compared to typical AOMS.^8-10^ At the opposite extremity of the age-at-onset spectrum, multiple sclerosis may occur in childhood or adolescence (paediatric-onset multiple sclerosis, POMS), which accounts for 2–10% of cases.^11^

RS FC studies have generally revealed a multifaceted pattern of increased and decreased RS FC within the major brain networks in multiple sclerosis patients compared to matched healthy controls (HC), with a balance between these phenomena varying according to disease severity and stage, suggesting a complex interplay between adaptive and maladaptive processes occurring throughout the course of the disease.^3,12,13^ Converging pieces of evidence also demonstrate that ageing exerts a crucial modulatory role in these processes. Studies in POMS have shown that functional network reorganization differs markedly between younger and AOMS patients, reflecting the distinct influences of ongoing brain maturation and ageing on multiple sclerosis-related damage.^14-16^ Similarly, longitudinal studies in healthy ageing and AOMS have shown progressive RS FC changes, reflecting a gradual shift from compensatory to inefficient network reorganization across the lifespan, supporting the notion that brain connectivity reorganization is a continuous and age-dependent process.^2,4,17,18^

Despite this background, no studies have specifically explored RS FC profiles in LOMS, nor whether advanced age at onset shapes these patterns differently from what observed in AOMS, or how they relate to clinical, cognitive and structural MRI measures. In light of this, we hypothesized that LOMS would show a distinct pattern of RS functional reorganization compared with AOMS, reflecting the influence of advanced age at onset on large-scale network abnormalities and their clinical, cognitive and structural MRI correlates.

By comparing RS FC within major large-scale brain networks of LOMS with a cohort of sex- and disease duration (DD)–matched AOMS, this study aimed to determine whether age at onset is associated with distinct patterns of functional network reorganization. Moreover, we aimed to clarify whether such alterations might be compatible with adaptive or maladaptive patterns in relation to clinical measures [Expanded Disability Status Scale (EDSS) score and DD], cognitive performance and structural MRI damage [T2 lesion volume (T2-LV) and normalized brain volume (NBV)].

## Materials and methods

### Standard protocol approvals, registrations and patient consents

Ethical approval was obtained from the IRCCS San Raffaele ethics committee (Protocol SENESCENT-MS), and all participants provided written informed consent in accordance with the Declaration of Helsinki.

### Subjects

From the database of the Neuroimaging Research Unit, IRCCS San Raffaele Scientific Institute (Milan, Italy), we retrospectively selected 203 multiple sclerosis patients (169 AOMS and 34 LOMS) acquired between May 2007 and January 2024. LOMS was defined as multiple sclerosis with clinical onset at age ≥50 years, in line with previous literature,^8,19^ whereas patients with clinical onset before 50 years were classified as AOMS. Multiple sclerosis onset was defined as the age at first appearance of multiple sclerosis-related clinical symptoms. Patients were included if they met the following criteria: (i) right-handedness (Edinburgh Handedness Inventory ≥33); (ii) no major systemic, psychiatric, neurological or other comorbid conditions other than multiple sclerosis; (iii) no history of drug or alcohol abuse; (iv) no contraindications to MRI (claustrophobia, pregnancy, breastfeeding); (v) relapse- and steroid-free for at least 3 months; (vi) stable multiple sclerosis treatment for at least 6 months; (vii) no use of medications potentially affecting RS FC (benzodiazepines, antiepileptic agents, anti-fatigue treatments).

We also selected 163 right-handed HC, divided into two age-based groups to match AOMS and LOMS (122 HC-AO, 41 HC-LO). HC underwent the same MRI protocol and served as reference for RS FC analyses. They had no neurological diseases or systemic disorders potentially affecting the central nervous system and had a normal neurological examination.

### Clinical and neuropsychological assessment

All multiple sclerosis patients underwent a complete neurological examination, with EDSS score rating, recording of DD, calculated from clinical onset to MRI assessment, and disease-modifying treatment (DMT) classified as moderate efficacy treatments [(MET): azathioprine, dimethyl fumarate, glatiramer acetate, interferon beta1a, teriflunomide] and high efficacy treatments [(HET): alemtuzumab, cladribine, fingolimod, natalizumab, ocrelizumab, rituximab, siponimod].

Moreover, all multiple sclerosis patients underwent an extensive neuropsychological assessment, administered by experienced neuropsychologists, using the Brief Repeatable Battery of Neuropsychological Tests (BRB-N).^20^ Specifically, the Selective Reminding Test (SRT) was used to evaluate verbal memory, the 10/36 Spatial Recall Test (SPART) was used to assess visual memory, the Symbol Digit Modalities Test (SDMT) and the Paced Auditory Serial Addition Test (PASAT) 2″ and 3″ per digit version were used to evaluate attention, and the Word List Generation test (WLG) was used to assess verbal fluency. *Z*-scores for all BRB-N tests were calculated using regression models accounting for sex, age and education, according to recently published Italian normative data.^21^ Domain-specific *Z*-scores were obtained by averaging test *Z*-scores within each domain, as previously described.^22^ Finally, the *Z*-score of global cognitive function (obtained by averaging *Z*-scores of cognitive domains) was calculated.^22^ Test failure was defined as a score at least 1.5 SD below the normative mean.^23^ Impairment in a cognitive domain was defined as failure in ≥1 BRB-N test assessing that domain, and global cognitive impairment as impairment in ≥2 domains.^23^ This classification was used for descriptive purposes only, and RS FC comparisons by cognitive status were not performed due to the small LOMS sample and limited statistical power.

### MRI acquisition

Within 48 h from the clinical and neuropsychological assessments, all participants underwent a single brain MRI session on two 3.0 T scanners [Intera (Scanner 1) and Ingenia CX (Scanner 2), Philips Medical Systems, Amsterdam, the Netherlands].

Scanner 1 included dual-echo (DE) turbo spin echo, high-resolution 3D T1-weighted fast field echo, and T2* echo-planar imaging (EPI) sequences.

Scanner 2 included sagittal 3D fluid-attenuated inversion recovery (FLAIR), high-resolution 3D T1-weighted magnetization-prepared rapid gradient-echo (MPRAGE), and T2* EPI sequences. During RS-fMRI, participants kept their eyes closed and avoided focused thoughts. RS-fMRI was acquired as a single run on both scanners, with a scan duration of 10 min on Scanner 1 and ∼8 min and 21 s on Scanner 2. Full acquisition parameters were provided in the Supplementary material.

### Structural MRI analysis

Brain T2-LV was quantified on DE sequence using a local thresholding segmentation technique (Jim 8.0, Xinapse Systems Ltd, Colchester, UK) for scans acquired on Scanner 1, or on 3D FLAIR/T1-weighted scans using a fully automated approach based on two 3D patch-wise convolutional neural networks^24^ for scans acquired on Scanner 2. Resulting lesion masks were carefully visually checked (and edited, if necessary) for each patient, and total T2-LV was calculated. After T1-hypointense lesion refilling, bias-field correction was performed within the SIENAx2 pipeline, and NBV was calculated on 3D T1-weighted scans using SIENAx2 software.^25^

### RS-fMRI preprocessing

fMRI data preprocessing was performed using CONN toolbox (https://web.conn-toolbox.org/).^26^ RS-fMRI images were realigned to the mean image, co-registered to the lesion-filled 3D T1-weighted scan, normalized to MNI space and smoothed with a 6-mm Gaussian kernel. Denoising included the first five aCompCor white matter (WM) and cerebrospinal fluid components,^27^ segmented on the lesion-filled and bias-field corrected 3D T1-weighted scans. The six motion parameters and their first temporal derivatives, ART-detected outliers, as well as spurious effects from the first two time points, were regressed out of the data. Linear detrending and band-pass filtering (0.01–0.1 Hz) were performed to partially remove low-frequency drifts and high-frequency physiological noise. As additional quality-control metrics, mean framewise displacement (FD) was computed from the six rigid-body motion parameters estimated during the realignment step. The temporal signal-to-noise ratio (tSNR) was defined as the mean signal intensity over time divided by the standard deviation of the RS-fMRI time series at each voxel. Mean tSNR was then calculated within a grey matter mask derived from the SPM12 grey matter probability map, thresholded at 0.25 and co-registered to the realigned RS-fMRI data.

### Independent component analysis

RS FC was assessed using independent component analysis (ICA) and the GIFT toolbox (http://mialab.mrn.org/software/gift/). ICA followed the standard three-step procedure of data reduction, group ICA, and back-reconstruction using spatio-temporal regression, as described in detail elsewhere.^28^ Forty group-level components were extracted using the minimum description length criterion, and decomposition stability was evaluated with ICASSO.^29^ Components reflecting motion or physiological noise were excluded based on spatial patterns, power spectra, and template matching. Ten networks were retained based on their spatial patterns and template matching: one sensorimotor network (SMN), one visual network (VN), one auditory network (AN), one thalamic network (TN), one cerebellar network (CN), one FPN lateralized to the right (R-FPN) and one lateralized to the left (L-FPN) hemisphere, one salience network (SN), one executive control network (ECN), and one default mode network (DMN). The spatial maps of the retained ICA components are provided in Supplementary Fig. 1. ComBat harmonization (https://github.com/Jfortin1/ComBatHarmonization) was applied to the subject-specific spatial maps of the retained networks produced by spatio-temporal regression using an Empirical Bayes approach with parametric adjustments.^30^ Scanner was included in the ComBat model to account for scanner-related variability, while sex, age and group membership (HC, AOMS, LOMS) were included as covariates of interest in order to preserve biologically relevant variance.

### Statistical analysis

Descriptive statistics of the groups were reported as means (SD) or median (interquartile range [IQR]) for continuous variables, while categorical variables were reported as frequencies. Demographic, clinical and neuropsychological data were compared between groups according to onset category (AO and LO) and/or disease status (HC and multiple sclerosis) using chi-square, two-sample *t* or Mann–Whitney tests, as appropriate. Sex- and scanner-adjusted linear models were applied to analyse NBV. Brain T2-LVs were compared using sex- and scanner-adjusted quantile regression. False discovery rate (FDR) correction was applied using the Benjamini–Hochberg procedure to account for multiple testing. RS-fMRI quality-control metrics, including mean FD and tSNR, were compared across HC-AO, AOMS, HC-LO and LOMS using scanner-adjusted linear models, and the onset-category × disease-status interaction effect was additionally tested. All analyses were conducted using SPSS v29, considering *P* < 0.05 as statistically significant.

Voxel-wise comparisons of RS FC within selected networks were performed using SPM12 software and second-level full factorial models including disease status (HC versus multiple sclerosis) and onset category (AO versus LO) as factors, with sex as a covariate. For each retained ICA network, second-level analyses were performed on the subject-specific back-reconstructed and ComBat-harmonized spatial maps. Within this framework, the following comparisons were assessed: (i) AOMS versus HC-AO and (ii) LOMS versus HC-LO. In the same models, we used an onset-category × disease-status interaction contrast to identify specific RS FC abnormalities for one patient group (i.e. LOMS or AOMS) relative to the other. All second-level SPM12 analyses within each network (group comparisons and interaction contrasts) were inclusively masked by the corresponding positive effects of interest of that network (thresholded at *P* < 0.05, family-wise error corrected for multiple comparisons, Supplementary Fig. 1) and tested at *P* < 0.001 (uncorrected at the voxel level, cluster extent threshold [*K*_E_] ≥ 10 voxels). This threshold was selected a priori as a spatial reporting criterion to reduce isolated voxel-level findings.^31^ As a sensitivity analysis, second-level SPM12 models were repeated including scanner as an additional covariate. This allowed us to evaluate whether the observed onset-category × disease-status interactions were consistent with those derived by models not adjusted for scanner. Additionally, *ad hoc* F-contrasts were created to specifically test for any residual effects of the scanner on RS FC, in order to determine whether such residual effects spatially overlapped with clusters of between-group difference.

For LOMS and AOMS, separately, average *Z*-scores of abnormal RS FC were extracted from clusters showing a significant onset-category × disease-status interaction using REX (www.nitrc.org/projects/rex). These *Z*-scores were then entered into Spearman’s rank correlation analysis to examine the relationship between RS FC abnormalities and clinical measures (EDSS and DD), cognitive performances in the different domains, and structural damage (T2-LV and NBV). For analyses involving structural MRI measures, scanner-adjusted partial Spearman’s rank correlations were used. No correction for multiple comparisons was applied. Therefore, these analyses should be considered exploratory. A *P* < 0.05 was considered statistically significant. As a sensitivity analysis, potentially influential observations were identified using Cook’s distance from linear models fitted to the ranked variables (Cook’s distance > 4/*n*). For structural MRI correlations, scanner was included in the corresponding models. When potentially influential observations were identified, the corresponding correlations were repeated after their exclusion. When more than one potentially influential observation was identified, analyses were performed both after individual exclusions and after simultaneous exclusion of all identified observations.

## Results

### Clinical, cognitive and structural MRI

Both AOMS and LOMS did not differ in terms of age, sex distribution and educational level compared to their respective HC-AO and HC-LO groups (all pFDR ≥ 0.098). AOMS and LOMS did not differ in terms of sex distribution, educational level and DD (all pFDR ≥ 0.053). Compared to AOMS, LOMS had higher EDSS score, higher prevalence of progressive phenotypes, and lower frequency of DMT use (all pFDR ≤ 0.016). Both multiple sclerosis groups had higher T2-LV and lower NBV compared to their corresponding HC groups (all pFDR < 0.001). A significant onset-category × disease-status interaction effect was observed only for T2-LV, with LOMS showing higher T2-LV compared to AOMS (pFDR = 0.028) (Table 1).

**Table 1:** Demographic, clinical and structural MRI features of the study groups

Variables	HC-AO (*N* = 122)	AOMS (*N* = 169)	HC-LO (*N* = 41)	LOMS (*N* = 34)	pFDR		
					AO: MS versus HC	LO: MS versus HC	MS: LOMS versus AOMS
**No. (%) of women/men**	58 (47.5%)/64 (52.5%)	100 (59%)/69 (41%)	25 (61%)/16 (39%)	15 (44%)/19 (56%)	0.098 ^a^	0.174 ^a^	0.159 ^a^
**Median age (IQR) [years]**	31.6 (27.5;41.0)	35.6 (30.0;40.1)	59.0 (55.3;62.6)	60.5 (56.6;66.1)	0.163 ^b^	0.188 ^b^	**0**.**006 ^b^**
**Median education (IQR) [years]**	14.0 (13.0;18.0)	13.0 (13.0;17.0)	13.0 (13.0;17.0)	13.0 (8.0;16.2)	0.107 ^b^	0.104 ^b^	0.053 ^b^
**Median age at onset (IQR) [years]**	—	27.0 (23.0;31.5)	—	53.0 (51.0;57.2)	—	—	**0**.**003 ^b^**
**Median DD (IQR) [years]**	—	6.0 (2.1;11.8)	—	4.5 (1.7;13.2)	—	—	0.894 ^b^
**Median EDSS (IQR)**	—	2.0 (1.0;3.5)	—	3.7 (1.5;6.0)	—	—	**0**.**016 ^b^**
**Clinical phenotype, No. (%)**							
**RRMS**	—	130 (77%)	—	17 (50%)	—	—	**0**.**008 ^a^**
**SPMS**		23 (14%)		5 (15%)			
**PPMS**		16 (9%)		12 (35%)			
**Ongoing treatment, No. (%) ^c^**							
**None**	—	37 (22%)	—	17 (50%)	—	—	**0**.**009 ^a^**
**MET**		79 (47%)		12 (35%)			
**HET**		53 (31%)		5 (15%)			
**Median T2-LV (IQR) [ml]**	0.00 (0.00;0.02)	2.92 (0.94;6.35)	0.13 (0.00; 0.48)	3.78 (1.75;9.35)	**<0**.**001 ^d^**	**<0**.**001 ^d^**	**0**.**028 ^d,e^**
**Mean NBV (SE) [ml]**	1597 (6)	1554 (5)	1546 (10)	1490 (11)	**<0**.**001** ^f^	**<0**.**001** ^f^	0.628 ^f,e^
**MRI scanner, No. (%) ^g^**							
**1**	68 (56%)	96 (57%)	23 (56%)	18 (53%)	0.856 ^a^	0.785 ^a^	0.679 ^a^
**2**	54 (44%)	73 (43%)	18 (44%)	16 (47%)			

Abbreviations: AOMS, adult-onset multiple sclerosis; DD, disease duration; EDSS, Expanded Disability Status Scale; FDR, false discovery rate; HC-AO, healthy controls matched to the adult-onset MS group; HC-LO, healthy controls matched to the late-onset MS group; HET, high efficacy treatment; IQR, interquartile range; LOMS, late-onset multiple sclerosis; MET, moderate efficacy treatment; No., number; RRMS, relapsing-remitting multiple sclerosis; SPMS, secondary progressive multiple sclerosis; PPMS, primary progressive multiple sclerosis. Bold is for statistically significant values.

^a^Chi-square test.

^b^Mann–Whitney U test.

^c^MET = azathioprine, dimethyl fumarate, glatiramer acetate, interferon beta1a, teriflunomide; HET, alemtuzumab, cladribine, fingolimod, natalizumab, ocrelizumab, rituximab, siponimod.

^d^Sex- and scanner-adjusted quantile regression.

^e^ *P*-value for significance of onset-category × disease-status interaction effect. *P*-values are false discovery rate corrected according to Benjamini–Hochberg procedure considering the overall number of tests performed.

^f^Sex- and scanner-adjusted linear models.

^g^Scanner 1 = Philips Intera; Scanner 2 = Philips Ingenia CX.

Table 2 shows the neuropsychological profile of AOMS and LOMS. Forty-seven (28%) AOMS and 16 (47%) LOMS were cognitively impaired. No significant between-groups differences emerged in terms of performance in cognitive domains nor in prevalence of impairment (all pFDR ≥ 0.235).

**Table 2:** Neuropsychological features of LOMS and AOMS patients

Cognitive domains	Score			No. of impaired (%)		
	AOMS (*n* = 169)	LOMS (*n* = 34)	LOMS versus AOMS pFDR	AOMS (*n* = 169)	LOMS (*n* = 34)	LOMS versus AOMS pFDR
**Mean *Z***	−0.41	−0.62	0.658 ^a^	49 (29%)	15 (44%)	0.257 ^b^
**Verbal memory**						
**Mean *Z***	−0.02	−0.10	0.703 ^a^	29 (17%)	4 (12%)	0.637 ^b^
**Visual memory**						
**Mean *Z***	−0.40	−0.55	0.515 ^a^	76 (45%)	16 (48%)	0.752 ^b^
**Attention**						
**Mean *Z***	−0.21	−0.58	0.569 ^a^	27 (16%)	10 (28%)	0.256 ^b^
**Verbal fluency**						
**Mean *Z***	−0.17	−0.29	0.645 ^a^	47 (28%)	16 (47%)	0.235 ^b^
**Global cognition**						

Abbreviations: AOMS, adult-onset multiple sclerosis; FDR, false discovery rate; LOMS, late-onset multiple sclerosis; No., number.

Bold is for statistically significant values. *P*-values are false discovery rate corrected according to Benjamini–Hochberg procedure considering the overall number of tests performed.

^a^Two-sample *t-*test.

^b^Chi-square test.

### RS FC findings

Results of RS FC analysis regarding sensorimotor (SMN), sensory (VN, AN), thalamic (TN), and cerebellar (CN) networks are reported in Table 3 and Fig. 1, while findings regarding high-order cognitive networks (R-FPN, L-FPN, SN, ECN, DMN) are reported in Table 4 and Fig. 2. RS FC abnormalities for AOMS and LOMS, separately (in relation to matched HC groups and highlighting onset-category × disease-status specific effects) are reported in the following paragraphs (*P* < 0.001 uncorrected, cluster extent ≥ 10).

**Figure 1:**
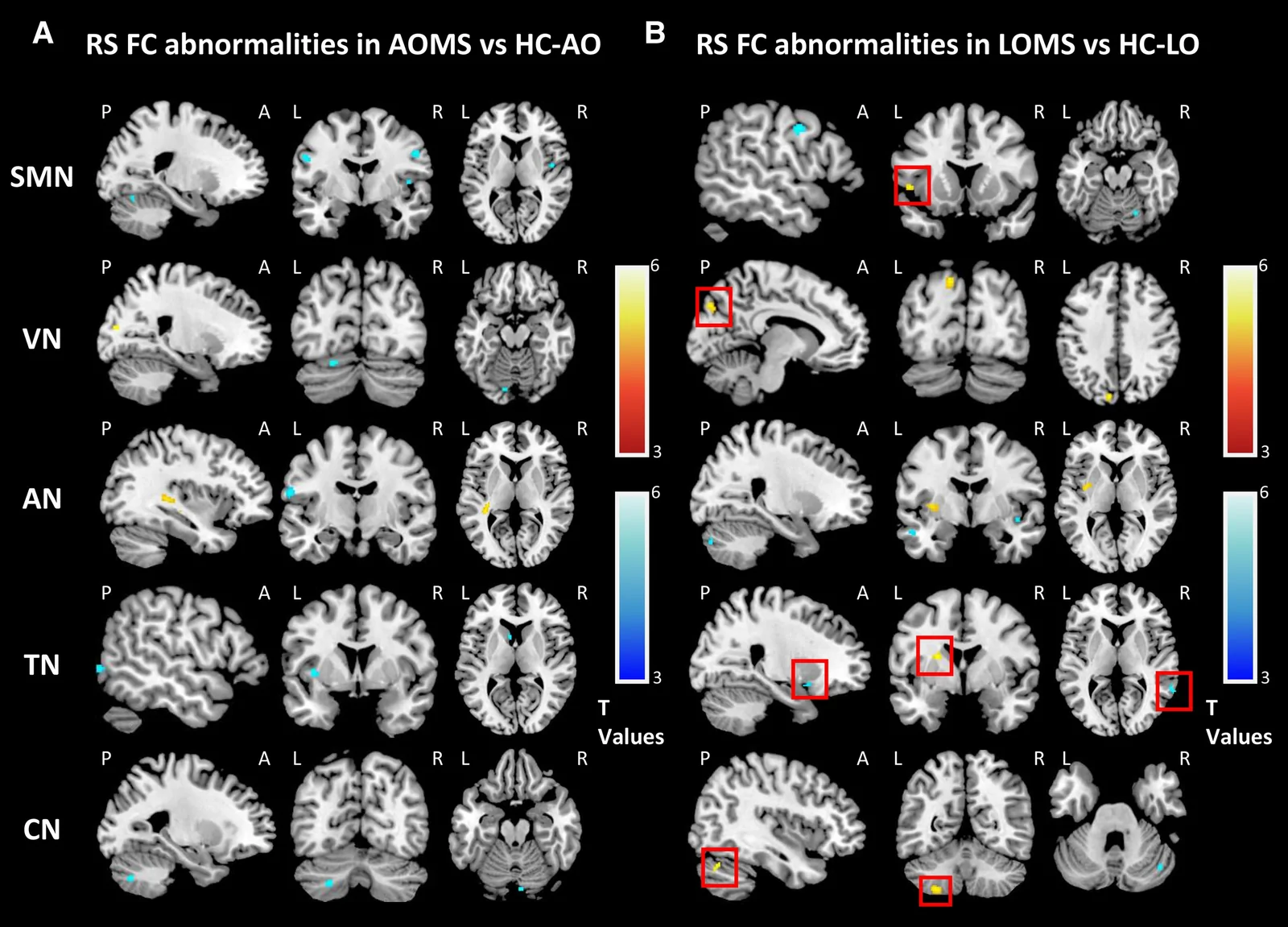
Abnormalities of RS FC of the selected main large-scale sensorimotor, sensory, thalamic, and cerebellar brain networks. (**A**) AOMS compared to HC-AO; (**B**) LOMS compared to HC-LO. Statistical test: SPM12 sex-adjusted full factorial model, significant at voxel-wise *P* < 0.001, uncorrected, cluster extent ≥10. Increased RS FC in MS versus HC is colour-coded in red-yellow, while decreased RS FC in MS versus HC is colour-coded in blue-lightblue. Red boxes indicate only those clusters within the displayed MS-versus-matched-HC contrasts that also showed a significant onset-category × disease-status interaction effect, indicating that the disease-related abnormality differed according to onset category. Sample sizes: adult-onset multiple sclerosis (*n* = 169), late-onset multiple sclerosis (*n* = 34), healthy controls matched to adult-onset group (*n* = 122), healthy controls matched to late-onset group (*n* = 41). Unit of analysis: participant. Abbreviations: A = anterior; AN = auditory network; AOMS = adult-onset multiple sclerosis; CN = cerebellar network; FC = functional connectivity; HC-AO = healthy controls matched to the adult-onset MS group; HC-LO = healthy controls matched to the late-onset MS group; L = left; LOMS = late-onset multiple sclerosis; *P* = posterior; R = right; RS = resting state; SMN = sensorimotor network; TN = thalamic network; VN = visual network.

**Figure 2:**
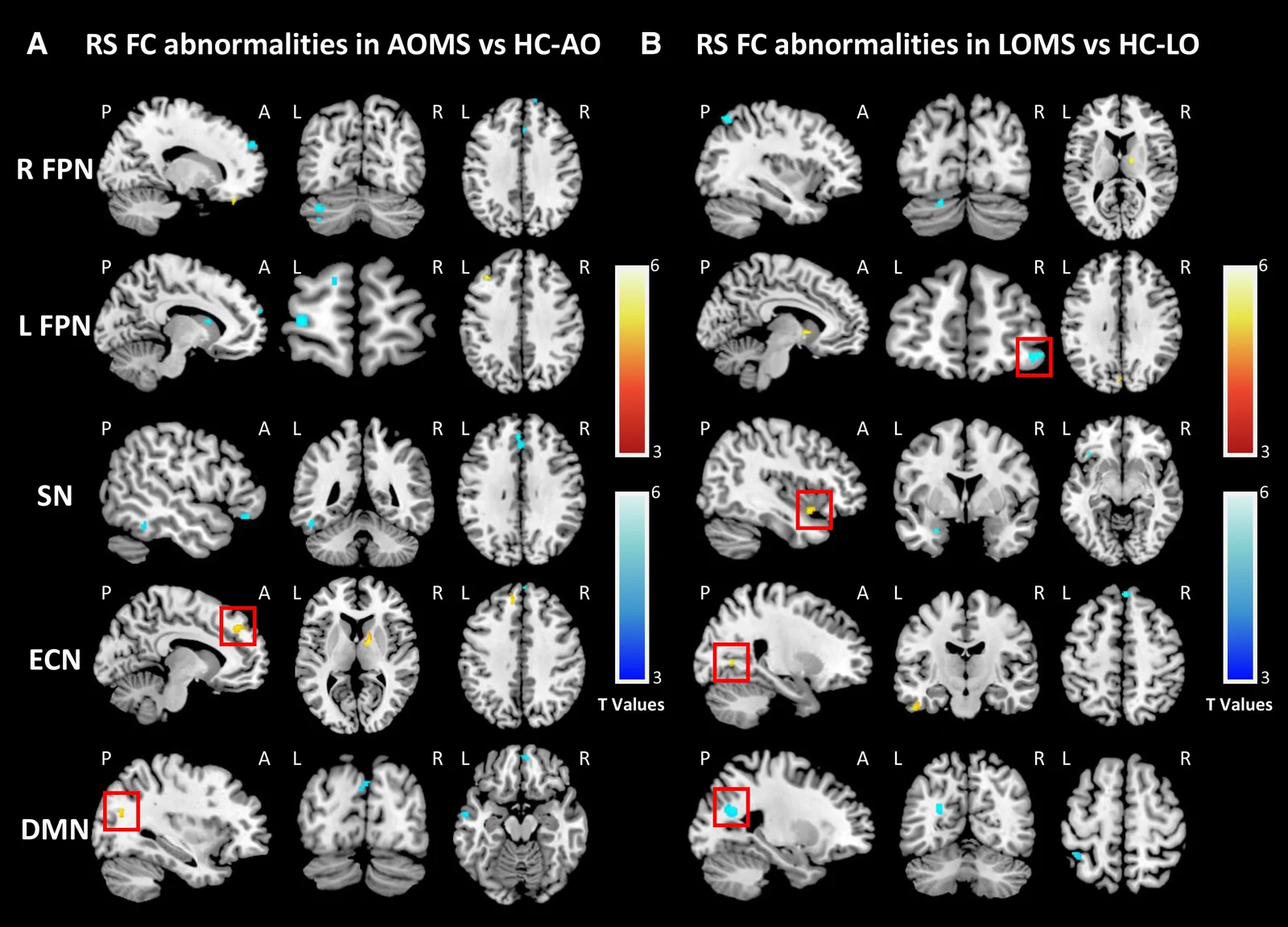
Abnormalities of RS FC of the selected main large-scale high-order cognitive brain networks. (**A**) AOMS compared to HC-AO; (**B**) LOMS compared to HC-LO. Statistical test: SPM12 sex-adjusted full factorial model, significant at voxel-wise *P* < 0.001, uncorrected, cluster extent ≥10. Increased RS FC in MS versus HC is colour-coded in red-yellow, while decreased RS FC in MS versus HC is colour-coded in blue-lightblue. Red boxes indicate only those clusters within the displayed MS-versus-matched-HC contrasts that also showed a significant onset-category × disease-status interaction effect, indicating that the disease-related abnormality differed according to onset category. Sample sizes: adult-onset multiple sclerosis (*n* = 169), late-onset multiple sclerosis (*n* = 34), healthy controls matched to adult-onset group (*n* = 122), healthy controls matched to late-onset group (*n* = 41). Unit of analysis: participant. Abbreviations: A = anterior; AOMS = adult-onset multiple sclerosis; DMN = default mode network; ECN = executive control network; FC = functional connectivity; FPN = fronto-parietal network; HC-AO = healthy controls matched to the adult-onset MS group; HC-LO = healthy controls matched to the late-onset MS group; L = left; LOMS = late-onset multiple sclerosis; *P* = posterior; R = right; RS = resting state; SN = salience network.

**Table 3:** Differences of RS FC in sensorimotor, sensory, thalamic, and cerebellar networks of AOMS and LOMS compared with the respective healthy control groups (i.e. HC-AO and HC-LO) (SPM12 sex-adjusted full factorial model, significant at voxel-wise *P* < 0.001, uncorrected, cluster extent [*K*_E_] ≥ 10)

Network	Comparison	Connected regions	MNI space coordinates (*x*, y, *z*)	*T-*value	*K*_E_
***RS FC abnormalities in AOMS* versus *HC-AO***					
Sensorimotor network	HC-AO > AOMS	R Postcentral gyrus	52 −4 34	4.00	24
		L Precentral gyrus	−54 −6 30	3.94	48
		R Insula	46 −6 8	3.86	19
		L Cerebellum (lobule VI)	−22 −66 −16	3.64	24
Visual network	HC-AO > AOMS AOMS > HC-AO	L Cerebellum (lobuleVI)	−20 −76 −20	3.47	14
		L Cerebellum (Crus I)	−12 −84 −16	3.43	11
		R SOG	22 −84 14	3.34	11
Auditory network	HC-AO > AOMS AOMS > HC-AO	L Postcentral gyrus	−60 −10 20	4.83	82
		L STG	−36 −36 6	4.43	65
Thalamic network	HC-AO > AOMS	L Precentral gyrus	−36 4 2	4.05	20
		L Caudate nucleus	−6 10 8	3.77	15
		L MOG	−54 −72 0	3.58	16
Cerebellar network	HC-AO > AOMS	L Cerebellum (Crus II)	−26 −70 −40	3.81	19
		R Cerebellum (Crus I)	6 −84 −20	3.78	14
***RS FC abnormalities in LOMS* versus *HC-LO***					
Sensorimotor network	HC-LO > LOMS LOMS > HC-LO	R Cerebellum (lobule VI)	22 −62 −18	3.76	10
		L Precentral gyrus	−56 −2 36	3.76	32
		**L IFG**	−48 14 0	4.10	18
Visual network	LOMS > HC-LO	**L Cuneus**	−10 −82 38	4.10	18
Auditory network	HC-LO > LOMS LOMS > HC-LO	R STG	44 0 −10	4.25	32
		R Cerebellum (Crus II)	24 −82 −34	3.88	10
		L MTG	−52 −4 20	3.62	11
		R MTG	70 −46 −4	4.23	20
		L Insula	−36 −6 4	3.89	33
Thalamic network	HC-LO > LOMS LOMS > HC-LO	**R MTG**	64 −50 6	4.43	56
		R Amygdala	30 −2 −16	4.07	13
		L MCC	−6 2 46	3.74	20
		**R Olfactory gyrus**	24 10 −14	3.74	19
		L Cerebellum (Crus II)	−30 −80 −38	3.72	14
		**L Caudate nucleus**	−24 0 18	3.81	20
Cerebellar network	HC-LO > LOMS LOMS > HC-LO	R Cerebellum (Crus I)	42 68 −30	3.53	12
		**L Cerebellum (lobule VIII)**	−28 −56 −54	4.37	73
		**R Cerebellum (Crus II)**	30 −76 −46	4.14	17
		L ITG	−64 −44 −18	3.83	10

The reported threshold (i.e. *P* < 0.001) applies to all listed regions. Clusters surviving the onset-category × disease-status interaction analysis are highlighted in bold. No correction for multiple comparisons was applied.

Abbreviations: AOMS, adult-onset multiple sclerosis; FC, functional connectivity; HC-AO, healthy controls matched to the adult-onset MS group; HC-LO, healthy controls matched to the late-onset MS group; IFG, inferior frontal gyrus; ITG, inferior temporal gyrus; *K*_E_, cluster extent; L, left; LOMS, late-onset multiple sclerosis; MCC, middle cingulate cortex; MOG, middle occipital gyrus; MTG, middle temporal gyrus; R, right; RS, resting state; SFG, superior frontal gyrus; SOG, superior occipital gyrus; STG, superior temporal gyrus.

**Table 4:** Differences of RS FC in high-order cognitive networks of AOMS and LOMS compared with the respective healthy control groups (i.e. HC-AO and HC-LO) (SPM12 sex-adjusted full factorial model, significant at voxel-wise *P* < 0.001, uncorrected, cluster extent [*K*_E_] ≥ 10)

Network	Comparison	Connected regions	MNI space coordinates (*x*, *y*, *z*)	*T-*value	*K*_E_
***RS FC abnormalities in AOMS* versus *HC-AO***					
Right fronto-parietal network	HC-AO > AOMS AOMS > HC-AO	L Cerebellum (Crus I)	−38 −70 −30	4.30	34
		R Medial SFG	14 58 32	4.09	53
		R IPL	36 −44 52	3.80	10
		L Cerebellum (Crus II)	−36 −68 −42	3.64	16
		R MCC	2 26 36	3.50	10
		R SFG	16 40 −24	3.82	31
Left fronto-parietal network	HC-AO > AOMS AOMS > HC-AO	L SFG	−26 62 4	4.35	21
		L Precentral gyrus	−44 4 54	4.01	18
		L Caudate nucleus	−12 12 14	3.98	16
		L SFG	−12 66 24	3.51	12
		L MFG	−38 32 38	3.68	11
Salience network	HC-AO > AOMS	L Medial SFG	−2 42 32	4.32	58
		L IFG	−52 34 −12	3.98	11
		L ITG	−50 −46 −18	3.86	22
Executive control network	HC-AO > AOMS AOMS > HC-AO	L Medial SFG	4 54 38	4.08	28
		R Cerebellum (Vermis)	2 −42 −10	3.79	30
		R Caudate nucleus	12 −2 8	4.44	41
		**R ACC**	2 56 0	4.30	19
		L Medial SFG	−10 40 40	3.93	33
Default mode network	HC-AO > AOMS AOMS > HC-AO	L MTG	−58 −6 −14	4.76	67
		R SFG	6 54 −16	4.02	23
		L Precuneus	2 −82 34	3.58	16
		**L MOG**	−36 −70 22	3.51	25
***RS FC abnormalities in LOMS* versus *HC-LO***					
Right fronto-parietal network	HC-LO > LOMS LOMS > HC-LO	L SPL	−36 −66 54	3.68	18
		L Cerebellum (Crus I)	−20 −78 −26	3.55	16
		R Thalamus	14 −8 12	3.60	14
Left fronto-parietal network	HC-LO > LOMS LOMS > HC-LO	**R MFG**	44 50 −10	3.61	13
		L Caudate nucleus	−8 8 0	4.25	14
		L Cuneus	−2 −80 32	3.92	10
Salience network	HC-LO > LOMS LOMS > HC-LO	L Amygdala	−26 2 −26	3.81	10
		L IFG	−40 26 −8	3.63	15
		**R Insula**	40 10 −14	4.57	28
Executive control network	HC-LO > LOMS LOMS > HC-LO	L Medial SFG	4 38 54	3.84	19
		**R Lingual gyrus**	24 −66 2	3.98	11
		L ITG	−48 −14 −32	3.64	21
Default mode network	HC-LO > LOMS	**L MOG**	−26 −62 24	4.61	81
		L SPL	−40 −44 60	3.64	16

The reported threshold (i.e. *P* < 0.001) applies to all listed regions. Clusters surviving the onset-category × disease-status interaction analysis are highlighted in bold. No correction for multiple comparisons was applied.

Abbreviations: ACC, anterior cingulate cortex; AOMS, adult-onset multiple sclerosis; FC, functional connectivity; HC-AO, healthy controls matched to the adult-onset MS group; HC-LO, healthy controls matched to the late-onset MS group; IFG, inferior frontal gyrus; IPL, inferior parietal lobule; ITG, inferior temporal gyrus; *K*_E_, cluster extent; L, left; LOMS, late-onset multiple sclerosis; MCC, middle cingulate cortex; MFG, middle frontal gyrus; MOG, middle occipital gyrus; MTG, middle temporal gyrus; R, right; RS, resting state; SFG, superior frontal gyrus; SPL, superior parietal lobule.

In summary, both AOMS and LOMS showed mixed patterns of RS FC decreases and increases across most networks, with largely overlapping regions of decreased RS FC. In AOMS, group-specific RS FC increases were limited to high-order cognitive networks, whereas LOMS exhibited more diffuse and extensive group-specific RS FC alterations involving both decreases and increases across nearly all networks. Finally, a partial divergent between groups of RS FC alteration was detected within the DMN.

#### RS FC abnormalities in sensorimotor, sensory, thalamic and cerebellar networks in AOMS (versus HC and compared to LOMS)

Compared with HC-AO, AOMS showed decreased RS FC within the SMN, involving the left pre- and right postcentral gyri, the right insula, and the left cerebellum. Within the VN, AOMS exhibited decreased RS FC in the left cerebellum compared to HC-AO, along with increased RS FC in the right superior occipital gyrus. Within the AN, AOMS showed decreased RS FC in left postcentral gyrus compared to HC-AO and increased RS FC in the left superior temporal gyrus. Within TN, decreased RS FC in left precentral gyrus, caudate nucleus and middle occipital gyrus was detected, while within CN, decreased RS FC in bilateral cerebellum was found. No significant onset-category × disease-status interaction effects were observed within sensorimotor, sensory, thalamic and cerebellar networks in AOMS (Table 3, Fig. 1).

#### RS FC abnormalities in sensorimotor, sensory, thalamic, and cerebellar networks in LOMS (versus HC and compared to AOMS)

Compared with HC-LO, within the SMN, LOMS showed decreased RS FC in the left precentral gyrus and right cerebellum and increased RS FC in the left inferior frontal gyrus, the latter being significant at onset-category × disease-status interaction analysis. With regards to the VN, LOMS showed increased RS FC versus HC-LO in the left cuneus, also significant at onset-category × disease-status interaction analysis. In the AN, mixed increased and decreased RS FC were identified in temporal regions, without being significant at onset-category × disease-status interaction analysis. Within TN, decreased RS FC in right amygdala, left middle cingulate cortex, right middle temporal gyrus and olfactory gyrus and increased RS FC in the left caudate nucleus were observed, the latter three RS FC changes were significant at the onset-category × disease-status interaction analysis. Regarding CN, limited decreased RS FC was found in the right cerebellum (Crus I), while increased RS FC, significant at onset-category × disease-status interaction analysis, in left and right cerebellum (left lobule VIII and right Crus II) was observed (Table 3, Fig. 1).

#### RS FC abnormalities in high-order cognitive networks in AOMS (versus HC and compared to LOMS)

AOMS exhibited decreased RS FC compared with HC-AO within the L- and R-FPN, involving superior frontal, parietal, middle cingulate, cerebellar and caudate regions. In both networks, circumscribed increased RS FC was also observed in frontal areas [right superior frontal gyrus in the R-FPN and left middle frontal gyrus (MFG) in the L-FPN]. AOMS also showed decreased RS FC within fronto-temporal regions of the SN. However, none of these high-order network RS FC abnormalities showed significant onset-category × disease-status interaction effects. Within the ECN, AOMS showed decreased RS FC in superior frontal and cerebellar regions, as well as increased RS FC in the right caudate nucleus and right anterior cingulate cortex, this latter showing a significant onset-category × disease-status interaction effect. Finally, within the DMN, AOMS exhibited decreased RS FC compared to HC-AO in fronto-temporal regions and left precuneus, while increased RS FC was detected in the left middle occipital gyrus, which was significant at onset-category × disease-status interaction analysis (Table 4, Fig. 2).

#### RS FC abnormalities in high-order cognitive networks in LOMS (versus HC and compared to AOMS)

LOMS exhibited decreased RS FC compared to HC-LO in several fronto-parietal regions within the L- and R-FPN, with RS FC decrease in the right MFG (L-FPN) also significant at onset-category × disease-status interaction analysis. Conversely, increased RS FC was found in the right thalamus within the R-FPN, while within the L-FPN increased RS FC was observed in the left caudate nucleus and cuneus, although not significant at the onset-category × disease-status interaction analysis. Within the SN, LOMS demonstrated decreased RS FC versus HC-LO in the left inferior frontal gyrus and amygdala and increased RS FC in the right insula, the latter being significant at onset-category × disease-status interaction analysis. In the ECN, LOMS showed decreased RS FC in the left medial superior frontal gyrus and increased RS FC in the left inferior temporal and right lingual gyri, with this latter also being significant at onset-category × disease-status interaction analysis. Finally, within the DMN, LOMS displayed decreased RS FC compared to HC-LO in the left superior parietal and left middle occipital gyri, with the latter also significant at onset-category × disease-status interaction analysis (Table 4, Fig. 2).

### Quality control and sensitivity analyses

Quality-control metrics are reported in the Supplementary material (Supplementary Table 1). Mean FD did not differ significantly between AOMS and HC-AO or between LOMS and HC-LO (*P* = 0.128 and *P* = 0.400, respectively), and no onset-category × disease-status interaction effect was observed (*P* = 0.954). Similarly, tSNR did not differ significantly between AOMS and HC-AO or between LOMS and HC-LO (*P* = 0.566 and *P* = 0.953, respectively), and no onset-category × disease-status interaction effect was observed (*P* = 0.836).

The overall pattern of onset-category × disease-status interactions derived from sex- and scanner-adjusted SPM12 models remained highly similar to that observed in the main analyses. Detailed results are reported in the Supplementary material (Supplementary Table 2). Scanner-related effects shown by the *ad hoc* F contrast were overall limited, although a relatively extensive cluster pattern was observed within the L-FPN. Importantly, these clusters did not spatially overlap with the main disease-related findings. Detailed results are reported in the Supplementary material (Supplementary Table 3).

### Exploratory correlation analysis

Overall, 117 exploratory associations were tested, including 18 associations in AOMS (2 group-specific clusters × 9 variables) and 99 associations in LOMS (11 group-specific clusters × 9 variables). These analyses were not corrected for multiple comparisons and are therefore reported as exploratory associations.

In AOMS, increased RS FC in the left middle occipital gyrus (DMN) was significantly associated with lower EDSS score (*ρ* = −0.20, 95% CI [−0.35; −0.05], *P* = 0.01). No other significant associations were found in AOMS.

In LOMS, significant associations were found between:

- higher EDSS score versus decreased RS FC in the right middle temporal gyrus (TN) (*ρ* = −0.39, 95% CI [−0.65; −0.05], *P* = 0.02);
- longer DD versus increased RS FC in the left caudate nucleus (TN) (*ρ* = 0.40, 95% CI [0.06; 0.66], *P* = 0.02);
- worse verbal memory (*ρ* = 0.38, 95% CI [0.02; 0.65], *P* = 0.03), verbal fluency (*ρ* = 0.45, 95% CI [0.06; 0.72], *P* = 0.02), and global cognition scores (*ρ* = 0.51, 95% CI [0.03; 0.74], *P* = 0.03) versus decreased RS FC in the right MFG (L-FPN);
- better visual memory (*ρ* = 0.40, 95% CI [0.05; 0.66], *P* = 0.02), and attention (*ρ* = 0.42, 95% CI [0.02; 0.70], *P* = 0.03) versus increased RS FC in the lobule VIII of the left cerebellum (CN);
- higher T2-LV versus decreased RS FC in the right middle temporal gyrus (TN) (*ρ* = −0.45, 95% CI [−0.70; −0.11], *P* = 0.01);
- lower NBV versus increased RS FC in the left inferior frontal gyrus (SMN) (*ρ* = −0.39, 95% CI [−0.65; −0.05], *P* = 0.03) and decreased RS FC in the right MFG (L-FPN) (*ρ* = 0.41, 95% CI [0.08; 0.67], *P* = 0.02).

Scatterplots illustrating all significant exploratory associations are provided in the Supplementary material (Supplementary Figs 2–6), showing both actual observed values for interpretability and rank-transformed values consistent with the Spearman correlation analyses. Sensitivity analyses showed that all associations identified in the original analysis retained their statistical significance after removing potentially influential data points (Supplementary Table 4).

## Discussion

By applying voxel-wise analysis to 10 large-scale networks identified with ICA, we characterized RS FC profiles in AOMS and LOMS and examined whether clinically defined age at onset was associated with different patterns of RS FC abnormalities. Both multiple sclerosis groups showed partly overlapping RS FC decreases and increases, but also group-specific RS FC abnormalities. AOMS showed RS FC increases in occipital DMN regions and in frontal regions of the ECN, whereas LOMS displayed diffuse RS FC alterations characterized by both increases and decreases across multiple large-scale networks. Exploratory uncorrected correlation analyses suggested associations between selected RS FC abnormalities and clinical, cognitive and structural MRI measures. These findings should be interpreted as hypothesis-generating, as they did not survive correction for multiple comparisons. Overall, these findings may be more consistent with a less efficient pattern of functional network reorganization in LOMS, in the context of older age, greater structural MRI damage, and a more severe clinical profile.

From a clinical point of view, LOMS showed an overall worse condition, with more severe disability, a higher prevalence of progressive phenotypes, and lower DMT exposure, a pattern consistent with the clinical and therapeutic complexity often reported in LOMS cohorts and with previous evidence of faster disability progression even when compared with AOMS matched for DD or age.^8,32-34^ Structurally, both multiple sclerosis groups exhibited lower NBV and higher T2-LV compared to their respective HC groups, but LOMS showed higher T2-LV compared with AOMS, in line with previous findings,^35^ suggesting a greater accumulation of WM damage.^36,37^

Regarding cognition, no significant between-group differences emerged in either domain-specific performance or impairment rates. Although this contrasts with previous reports of worse cognitive profiles in LOMS,^9,10^ limited statistical power, due to the small LOMS sample and FDR correction, may have hindered detection of subtle differences. The trend towards lower mean *Z*-scores and higher impairment rates across domains nonetheless suggests potentially worse cognitive functioning in LOMS. Variability across studies may also reflect cognitive function not included in the BRB-N (e.g. social cognition) or differences in the cognitive batteries and normative data used.^9,10^

RS FC analysis revealed diffuse abnormalities in both multiple sclerosis groups compared with HC, involving nearly all networks and consistent with previous evidence of widespread disruption in multiple sclerosis.^13^ Both AOMS and LOMS showed overlapping regions of decreased and increased RS FC, together with distinct group-specific alterations. Onset-category × disease-status interaction effects highlighted these differences: AOMS showed limited RS FC increases, mainly in the right anterior cingulate cortex within the ECN, whereas LOMS exhibited more heterogeneous alterations, with decreases and diffuse increases across several networks. A divergent pattern also emerged within the DMN, with increased RS FC in AOMS and decreased RS FC in LOMS in the left middle occipital gyrus. In AOMS, these circumscribed frontal–occipital increases may reflect early adaptive reorganization within functionally preserved networks,^12,38^ particularly within posterior DMN regions.^39^ In LOMS, the widespread changes may instead reflect less efficient reorganization, possibly related to age-associated energetic costs, hyperconnectivity and dedifferentiated recruitment, as described in ageing and in elderly multiple sclerosis with high disability.^3,4,36^ The higher prevalence of primary progressive phenotype in LOMS may also contribute to DMN FC decreases, consistent with prior evidence.^40^ Overall, these findings suggest that advanced age at onset may influence network reorganization in multiple sclerosis, with age-related RS FC changes potentially reducing the capacity to sustain efficient adaptive mechanisms.^4,36,41^

In line with network-level findings, the exploratory uncorrected correlation analyses suggested potentially different association patterns between RS FC abnormalities and clinical, cognitive and structural MRI measures in AOMS and LOMS. In AOMS, increased RS FC in the left middle occipital gyrus within the DMN was associated with lower EDSS in exploratory, uncorrected analyses. This finding should not be interpreted as evidence of a direct causal link between occipital DMN connectivity and disability. Rather, it may indicate that patients with lower disability and, by inference, lower global disease burden retain greater functional reserve. Posterior DMN regions, including occipital and precuneal areas, may support compensatory redistribution of network activity when structural damage is still relatively limited.^39^ Under this hypothesis, increased posterior DMN RS FC in less disabled AOMS patients may reflect adaptive network reorganization maintaining functional integration despite multiple sclerosis-related injury. With increasing disability and structural damage, however, this compensatory capacity may become insufficient or inefficient, consistent with evidence that FC increases in multiple sclerosis may be adaptive at some disease stages but maladaptive or energetically costly in more advanced disease.^3,13,42^ The younger age and relatively preserved functional status of AOMS patients may contribute to maintaining an efficient network configuration despite structural damage.^13,43^ Conversely, in LOMS, several RS FC abnormalities were exploratively associated with worse disability, poorer cognitive performance and greater structural MRI damage, a pattern that may be compatible with a less efficient pattern of functional reorganization in this group. Decreased RS FC of the right MFG within the L-FPN showed exploratory associations with lower NBV and worse cognitive scores, aligning with the established role of the L-FPN in executive and attentional control^44^ and with evidence of its involvement in multiple sclerosis-related cognitive inefficiency.^3,12,45^ Longer DD was exploratively associated with increased RS FC in the left caudate nucleus within the TN, whereas decreased RS FC within the middle temporal gyrus of the same network showed exploratory associations with higher EDSS and higher T2-LV, aligning with evidence implicating thalamo-cortical disruption in multiple sclerosis disability.^46^ The compensatory capacity observed in AOMS may be attenuated in LOMS, although the exploratory nature of these associations does not allow firm conclusions about whether RS FC increases reflect inefficient overactivation rather than effective adaptation, or whether RS FC decreases indicate network disconnection.^3,13,36,42,47^ This pattern may be compatible with the concept of network collapse, whereby age- and multiple sclerosis-related mechanisms (demyelination, synaptic inefficiency, reduced plasticity), together with structural and clinical burden, may limit functional reorganization.^3,12,36,48^ However, the associations within the CN with better cognitive functioning could reflect residual adaptive mechanisms in this group, consistent with evidence linking cerebellar connectivity to compensatory reorganization in multiple sclerosis.^45,49^

Our study has some limitations. The relatively small LOMS group reduced statistical power, increased inter-subject variability, and may partly explain the lack of clusters surviving multiple-comparison correction, although it reflects the epidemiological rarity of the phenotype. The cross-sectional design does not allow evaluation of the temporal evolution of RS FC changes nor causal inference on age-at-onset effects. Accordingly, these findings should be interpreted as differences between clinically defined onset categories within an age-matched HC framework, rather than as definitive evidence of a pure or independent age-at-onset effect. In addition, the more severe clinical profile of LOMS, including higher disability, greater structural disease burden, higher prevalence of progressive phenotypes, and lower DMT exposure, may have contributed to the observed RS FC abnormalities. We also cannot exclude that delayed recognition of early symptoms, delayed diagnosis, or a longer subclinical or prodromal disease phase may have contributed to the greater lesion burden and worse clinical status observed in LOMS. Although ComBat harmonization reduces scanner-related variability, residual effects related to acquisition differences cannot be fully excluded. However, sensitivity analyses including scanner as an additional covariate yielded largely unchanged findings, and clusters showing significant residual scanner-related effects did not spatially overlap with the main disease-related results. In addition, RS-fMRI was acquired in a single run, and scan duration slightly differed between scanners, which may have influenced robustness of RS FC estimates. On the other hand, although RS-fMRI scan duration was not particularly long, it exceeded the minimum recommended length for reliable estimation of basic RS FC metrics,^50^ while remaining short enough to avoid the practical difficulties that longer scans could pose in clinical settings. Moreover, the use of the BRB-N limits the assessment of executive and social cognitive functions, which may differ between AOMS and LOMS. Finally, the exploratory uncorrected correlation analyses should be interpreted cautiously, given that such correlations did not survive correction for multiple comparisons and may include false positive findings. Future longitudinal studies with larger LOMS samples, integrating structural, functional and cognitive measures, are needed to clarify trajectories of functional reorganization and to identify early rehabilitative targets.

In conclusion, our results suggest that LOMS is associated with a distinct pattern of functional reorganization compared with AOMS within a cross-sectional, age-matched HC framework. The limited RS FC increase observed within posterior DMN regions in AOMS possibly reflects adaptive mechanisms. In contrast, LOMS exhibited diffuse RS FC increases and decreases across several networks, which may reflect less efficient reorganization, as they showed exploratory associations with more severe disability, worse cognitive performance, higher lesion load and brain atrophy. Nevertheless, selective RS FC increase within CN was exploratively associated with better cognitive performance, suggesting that residual adaptive mechanisms may coexist with a broader pattern of less efficient functional reorganization. Overall, these findings may be compatible with a less efficient pattern of functional reorganization in LOMS, potentially related to the combined effects of multiple sclerosis- and age-related factors.

## Supplementary Material

fcag396_Supplementary_Data

## Data Availability

The anonymized dataset used and analysed during the current study is available from the corresponding author upon reasonable request.
