# Supplementary material for: Advanced age at onset is associated with distinct functional brain reorganization in multiple sclerosis
Source: Brain Commun. 2026 Oct 16;8(5):fcag396. doi: 10.1093/braincomms/fcag396 (PMC13714548; doi:10.1093/braincomms/fcag396)
Supplement: Supplementary file 1 [file fcag396_supplementary_data.pdf]

Supplementary Methods

This section provides detailed parameters for MRI acquisition.

MRI acquisition

MRI data were acquired in 68 (56%) HC-AO, 96 (57%) AOMS, 23 (56%) HC-LO, and 18 (53%) LOMS participants on Scanner 1, and in 54 (44%) HC-AO, 73 (43%) AOMS, 18 (44%) HC-LO, and 16 (47%) LOMS participants on Scanner 2.

For scanner 1 (Philips Intera):

- Axial dual-echo turbo spin echo (field of view [FOV]=240×240 mm; 44 slices, 3-mm thick, matrix=256×256, pixel size 0.94 × 0.94 mm, repetition time [TR]=2599 ms, echo time [TE]=16-80 ms, echo train length [ETL]=6, flip angle=90°);
- Axial 3D T1-weighted fast field echo (FOV=230×230 mm, 220 slices, 0.8-mm thick, matrix=256×256 mm, pixel size= 0.89×0.89 mm, TR=25 ms, TE=4.6 ms, flip angle=30°);
- T2*-weighted echo planar imaging sequence (FOV=240×240 mm², 30 axial slices, 4 mm thick, voxel size=1.87×1.87×4 mm³, TR=3000 ms, TE=35 ms, flip angle=90°, and 200 volumes).

For Scanner 2 (Philips Ingenia CX):

- Sagittal 3D FLAIR (FOV=256×256 mm, 192 slices, voxel size=1×1×1 mm, matrix=256×256 mm, TR=4800 ms, TE=268 ms, inversion time [TI]=1650 ms, ETL=167, flip angle= 40°);
- Sagittal 3D T1-weighted magnetization-prepared rapid gradient-echo (FOV=256×256 mm, 204 slices, 1-mm thick,matrix= 256×256 mm, pixel size= 1×1 mm, TR=7 ms, TE=3.2 ms, TI= 1000 ms, flip angle= 8°);

- T2*-weighted echo planar imaging sequence (FOV=240×240 mm^2, 48 axial slices, 3 mm thick, voxel size=2.5×2.5×3 mm^3, TR=1567 ms, TE=35 ms, flip angle=70°, and 320 volumes).

Supplementary Table 1. Mean values of quality control metrics assessed on resting state functional MRI data of study subjects.

Variables	HC-AO (N=122)	AOMS (N=169)	HC-LO (N=41)	LOMS (N=34)	p value		
					AO: MS *vs* HC	LO: MS *vs* HC	MS: LOMS *vs* AOMS[b]
**Mean FD (SE)**	0.14 (0.01)	0.16 (0.01)	0.20 (0.01)	0.22 (0.01)	0.128[a]	0.400[a]	0.954[a]
**Mean tSNR (SE)**	53.10 (0.8)	52.52 (0.7)	51.18 (1.4)	51.07 (1.4)	0.566[a]	0.953[a]	0.836[a]

[a]Scanner-adjusted linear models; [b]p-value for significance of onset-category × disease-status interaction effect

Abbreviations: AOMS=adult-onset multiple sclerosis; FD=framewise displacement; HC-AO=healthy controls matched to the adult-onset MS group; HC-LO=healthy controls matched to the late-onset MS group; SE=standard error; tSNR=temporal signal-to-noise ratio.

Supplementary Table 2. Clusters showing significant onset-category × disease-status interaction effects on RS FC across the ICA-retained networks in the sensitivity analysis including scanner as an additional covariate (SPM12 sex- and scanner-adjusted full factorial model, significant at voxel-wise $p<0.001$, uncorrected, cluster extent [Ke] ≥10). No correction for multiple comparisons was applied.

Network	Comparison	Connected regions	MNI space coordinates (x, y, z)	T value	K_E
Sensorimotor Network	LOMS > HC-LO	L IFG	-48 14 0	4.09	18
Visual Network	LOMS > HC-LO	L Cuneus	-10 -82 38	4.37	39
Auditory Network	-	-	-	-	-
Thalamic Network	HC-LO > LOMS	R MTG	64 -50 6	4.42	55
		R Olfactory gyrus	24 10 -14	3.73	19
	LOMS > HC-LO	L Caudate Nucleus	-24 0 18	3.80	20
Cerebellar Network	LOMS > HC-LO	L Cerebellum (Lobule VIII)	-28 -56 -54	4.36	73
		R Cerebellum (Crus II)	30 -76 -46	4.15	18
Right Fronto-parietal Network	-	-	-	-	-
Left Fronto-parietal Network	HC-LO > LOMS	R MFG	44 50 -10	3.60	13
Salience Network	LOMS > HC-LO	R Insula	40 10 -14	4.57	29
Executive Control Network	AOMS > HC-AO	R ACC	2 56 0	4.29	19
	LOMS > HC-LO	R Lingual Gyrus	24 -66 2	3.97	11
Default Mode Network	AOMS > HC-AO	L MOG	-36 -70 22	3.47	25
	HC-LO > LOMS	L MOG	-26 -62 24	4.55	82

Abbreviations: ACC=anterior cingulate cortex; AOMS=adult-onset multiple sclerosis; FC=functional connectivity; HC-AO=healthy controls matched to the adult-onset MS group; HC-LO=healthy controls matched to the late-onset MS group; ICA=independent component analysis; IFG=inferior frontal gyrus; Ke=cluster extent; L=left; LOMS=late-onset multiple sclerosis; MFG=middle frontal gyrus; MOG=middle occipital gyrus; MTG=middle temporal gyrus; R=right; RS=resting state.

Supplementary Table 3. Main effect of scanner on RS FC across the ICA-retained networks in the sensitivity analysis including scanner as an additional covariate (SPM12 sex- and scanner-adjusted full factorial model, significant at voxel-wise p<0.001, uncorrected, cluster extent [Ke] ≥10).

**Network**	**Brain regions**	**MNI space coordinates (x, y, z)**	**F value**	**K_E**
Sensorimotor Network	L Cerebellum (Lobule VIII)<br>R Cerebellum (Lobule VIII)	-12 -66 -52<br>12 -68 -52	19.04<br>27.63	25 59
Visual Network	-	-	-	-
Auditory Network	-	-	-	-
Thalamic Network	-	-	-	-
Cerebellar Network	R Cerebellum (Lobule IX)	2 -46 -40	20.85	42
Right Fronto-parietal Network	-	-	-	-
Left Fronto-parietal Network	L IFG<br>R IPL<br>L ITG	-46 46 -12<br>58 -50 50<br>-58 -34 -20	26.88<br>23.94<br>18.41	267<br>43<br>57
Salience Network	-	-	-	-
Executive Control Network	-	-	-	-
Default Mode Network	-	-	-	-

Abbreviations: FC=functional connectivity; ICA=independent component analysis; IFG=inferior frontal gyrus; IPL=inferior parietal lobule; ITG=inferior temporal gyrus; Ke=cluster extent; L=left; R=right; RS=resting state.

Supplementary Table 4. Sensitivity analysis of statistically significant exploratory correlations after exclusion of potentially influential observations identified using Cook's distance. Original correlation coefficients and p-values are shown alongside those obtained after exclusion of potentially influential observations, where identified.

**Group**	**Association**	**Original ρ**	**Original p**	**No. excluded**	**ρ after exclusion**	**p after exclusion**
AOMS	EDSS – left MOG (DMN)	−0.20	0.01	4	−0.27	<0.01
LOMS	Disease duration – left caudate (TN)	0.40	0.02	1	0.49	<0.01
LOMS	EDSS – right MTG (TN)	−0.39	0.02	1	−0.47	0.01
LOMS	Verbal memory – right MFG (L-FPN)	0.38	0.03	2	0.58	<0.01
LOMS	Verbal fluency – right MFG (L-FPN)	0.45	0.02	1	0.52	<0.01
LOMS	Global cognition – right MFG (L-FPN)	0.51	0.03	1	0.64	<0.01
LOMS	Visual memory – left lobule VIII (CN)	0.40	0.02	1	0.50	<0.01
LOMS	Attention – left lobule VIII (CN)	0.42	0.03	2	0.54	<0.01
LOMS	T2-LV – right MTG (TN)[a]	−0.45	0.01	0	—	—
LOMS	NBV – left IFG (SMN)[a]	−0.39	0.03	1	−0.51	<0.01
LOMS	NBV – right MFG (L-FPN)[a]	0.41	0.02	2	0.60	<0.01

[a]Structural MRI associations were assessed using scanner-adjusted partial Spearman's rank correlations.
For the T2-LV–right MTG association in the LOMS group, no potentially influential observation was identified; therefore, no exclusion analysis was performed.
For associations with more than one potentially influential observation, the table reports the results after simultaneous exclusion of all identified observations. Results after individual exclusions were:

- EDSS–left MOG in the AOMS group: ρ = −0.22, p<0.01 for each of the four individual exclusions;
- Verbal memory–right MFG in the LOMS group: ρ = 0.51, p<0.01 and ρ=0.44, p=0.01;
- Attention–left lobule VIII in the LOMS group: ρ=0.48, p<0.01 and ρ=0.48, p<0.01;
- NBV–right MFG in the LOMS group: partial ρ=0.51, p<0.01 and partial ρ=0.47, p<0.01.

Abbreviations: AOMS=adult-onset multiple sclerosis; CN=cerebellar network; DMN=default mode network; EDSS=Expanded Disability Status Scale; IFG=inferior frontal gyrus; L-FPN=left fronto-parietal network; LOMS=late-onset multiple sclerosis; MFG=middle frontal gyrus; MOG=middle occipital gyrus; MTG=middle temporal gyrus; NBV=normalized brain volume; No.=number; SMN=sensorimotor network; T2-LV=T2 lesion volume; TN=thalamic network.

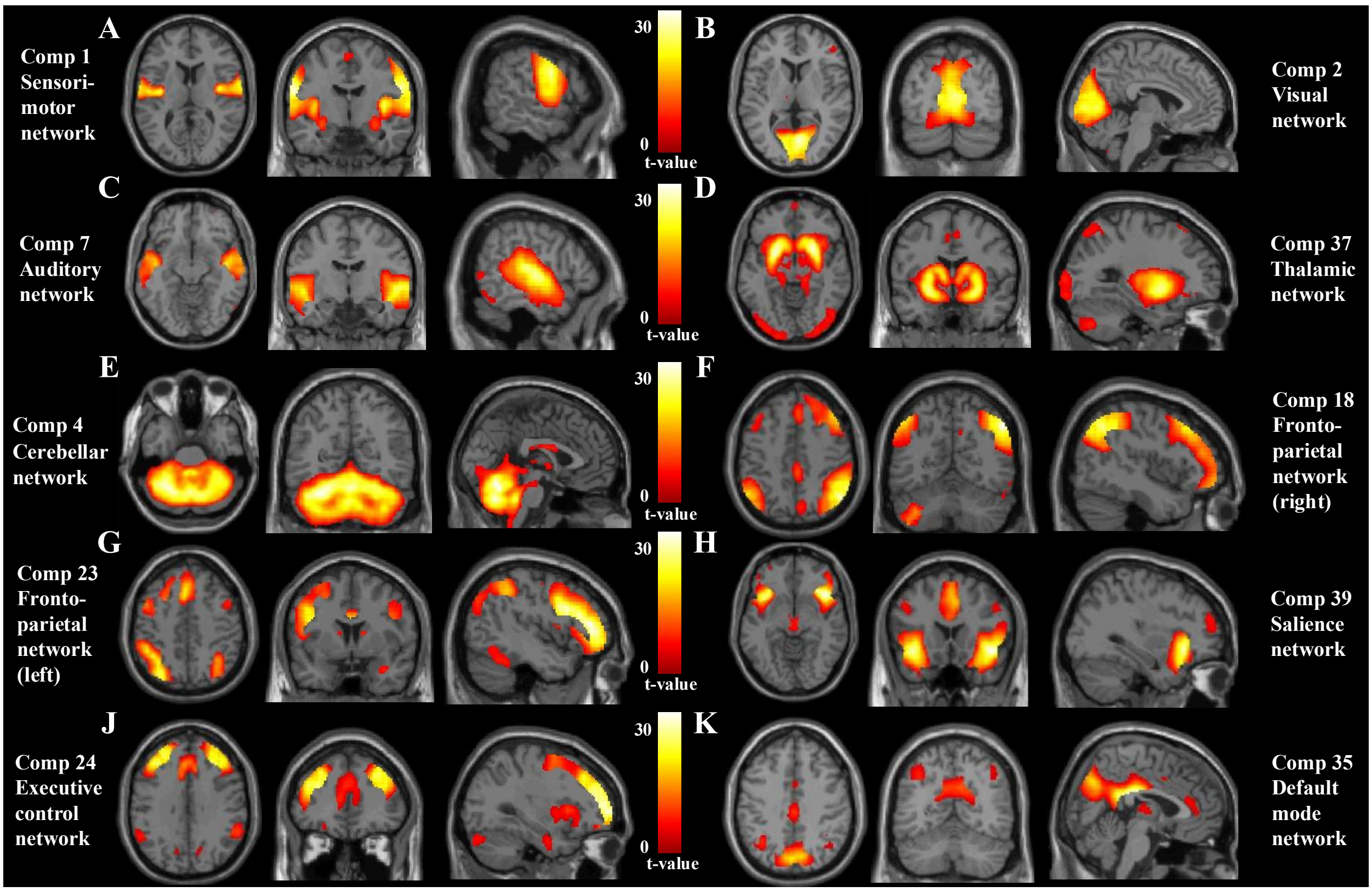

Supplementary Figure 1. Spatial patterns of the 10 ICA-derived networks included in the analysis, which were also used as inclusive masks for second-level comparisons (SPM12 sex-adjusted full factorial model, positive effects of interest significant at voxel-wise p<0.05, family-wise error corrected for multiple comparisons): sensorimotor network (A), visual network (B), auditory network (C), thalamic network (D), cerebellar network (E), right fronto-parietal network (F), left fronto-parietal network (G), salience network (H), executive control network (J), and default mode network (K). Networks were retained after exclusion of artefactual ICA components based on spatial patterns, power spectra, and template matching. Maps are displayed on the SPM canonical single-subject T1 template in MNI space. Group-level voxel-wise analyses reported in Figures 1–2 were performed within these network masks on the back-reconstructed subject-specific spatial maps. Unit of analysis: participant.

Abbreviations: Comp=component; ICA=independent component analysis.

EDSS – Left MOG (DMN)

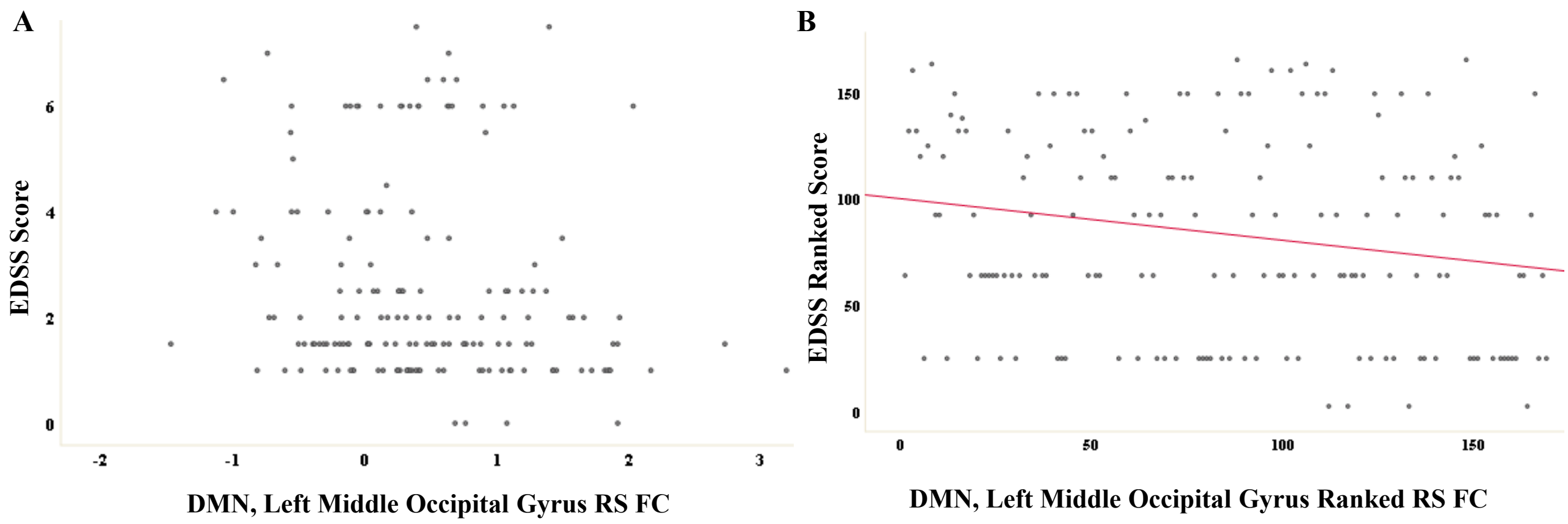

Supplementary Figure 2. Scatterplots illustrating the association between EDSS and RS FC in the left MOG within the DMN in the AOMS cohort. Panel A shows actual observed values to display the underlying data distribution, whereas Panel B shows rank-transformed data consistent with the Spearman correlation analysis. The red line in Panel B represents the linear fit for visualization purposes only. Each datapoint represents one patient. In exploratory analyses, lower EDSS was associated with increased RS FC in the left MOG ($\rho = –0.203$, 95% CI [-0.35; -0.05], $p = 0.01$). Sensitivity analysis excluding potentially influential observations identified using Cook's distance showed that all associations identified in the original analysis retained their statistical significance (Supplementary Table 4).

Abbreviations: AOMS=adult-onset multiple sclerosis; CI=confidence interval; DMN=default mode network; EDSS=Expanded Disability Status Scale; FC=functional connectivity; MOG=middle occipital gyrus; RS=resting state.

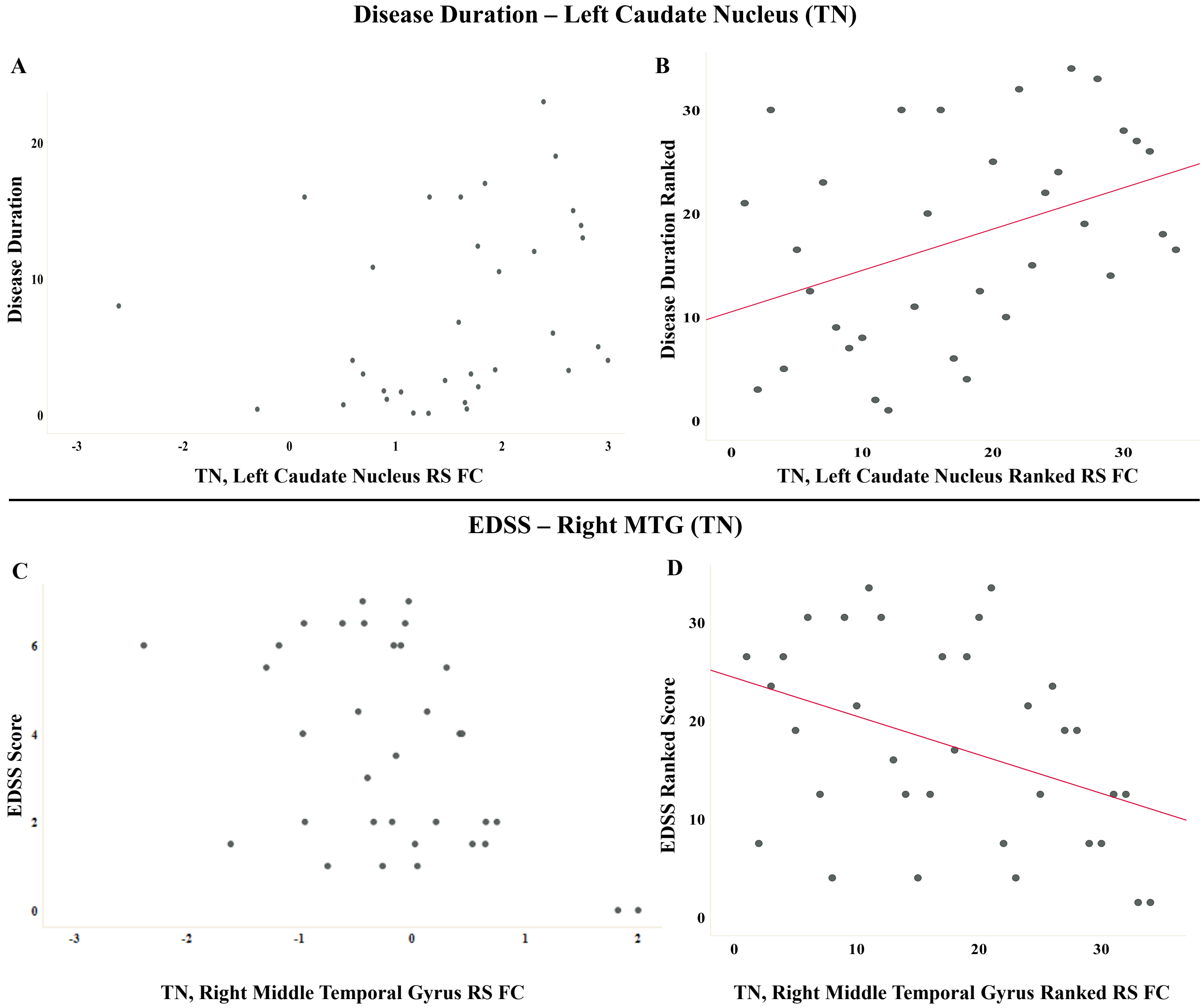

Supplementary Figure 3. Scatterplots illustrating the associations between clinical measures and RS FC abnormalities within the TN in the LOMS cohort. Panels A and B show the association between disease duration and RS FC in the left caudate nucleus, whereas Panels C and D show the association between EDSS and RS FC in the right MTG. Panels A and C display actual observed values to show the underlying data distribution, whereas Panels B and D display rank-transformed data consistent with the Spearman correlation analyses. The red lines in Panels B and D represent the linear fit for visualization purposes only. Each datapoint represents one patient. In exploratory analyses, longer disease duration was associated with increased RS FC in the left caudate nucleus ($\rho = 0.40$, 95% CI [0.06; 0.66], $p = 0.02$), while higher EDSS was associated with decreased RS FC in the right MTG ($\rho = -0.39$, 95% CI [-0.65; -0.05], $p = 0.02$). Sensitivity analysis excluding potentially influential observations identified using Cook's distance showed that all associations identified in the original analysis retained their statistical significance (Supplementary Table 4).

Abbreviations: CI=confidence interval; EDSS=Expanded Disability Status Scale; FC=functional connectivity; LOMS=late-onset multiple sclerosis; MTG=middle temporal gyrus; RS=resting state; TN=thalamic network.

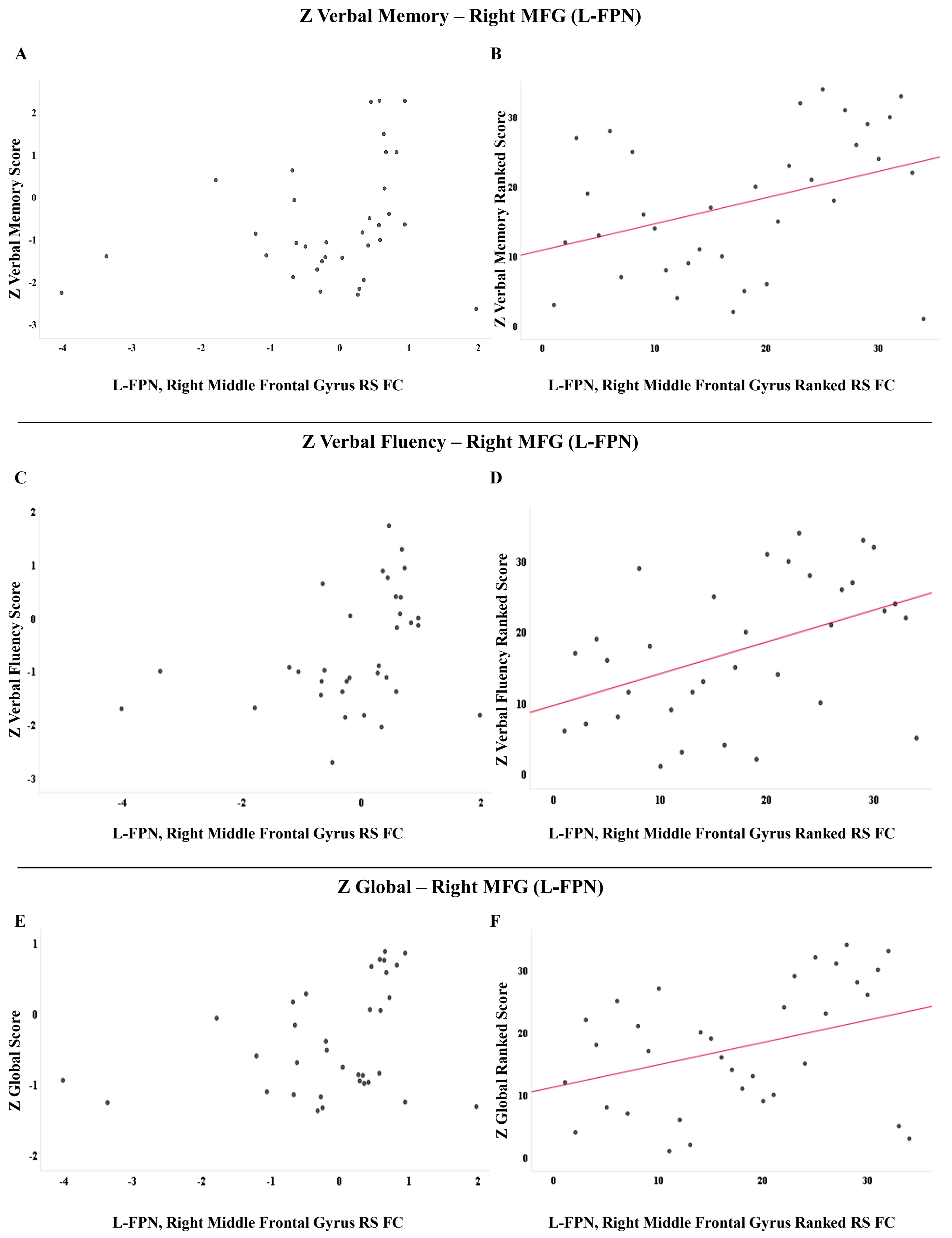

Supplementary Figure 4. Scatterplots illustrating the associations between cognitive domain z-scores and RS FC abnormalities within the L-FPN in the LOMS cohort. Panels A and B show the association between verbal memory and RS FC in the right MFG, Panels C and D show the

association between verbal fluency and RS FC in the right MFG, and Panels E and F show the association between global cognition and RS FC in the right MFG. Panels A, C, and E display actual observed values to show the underlying data distribution, whereas Panels B, D, and F display rank-transformed data consistent with the Spearman correlation analyses. The red lines in Panels B, D, and F represent the linear fit for visualization purposes only. Each datapoint represents one patient. In exploratory analyses, worse verbal memory ($\rho = 0.38$, 95% CI [0.02; 0.65], $p = 0.03$), verbal fluency ($\rho = 0.45$, 95% CI [0.06; 0.72], $p = 0.02$), and global cognition ($\rho = 0.51$, 95% CI [0.03; 0.74], $p = 0.03$) were associated with decreased RS FC in the right MFG. Sensitivity analysis excluding potentially influential observations identified using Cook's distance showed that all associations identified in the original analysis retained their statistical significance (Supplementary Table 4).

Abbreviations**:** CI=confidence interval**;** FC=functional connectivity; L-FPN=left fronto-parietal network; LOMS=late-onset multiple sclerosis; MFG=middle frontal gyrus; RS=resting state; Z=z-score.

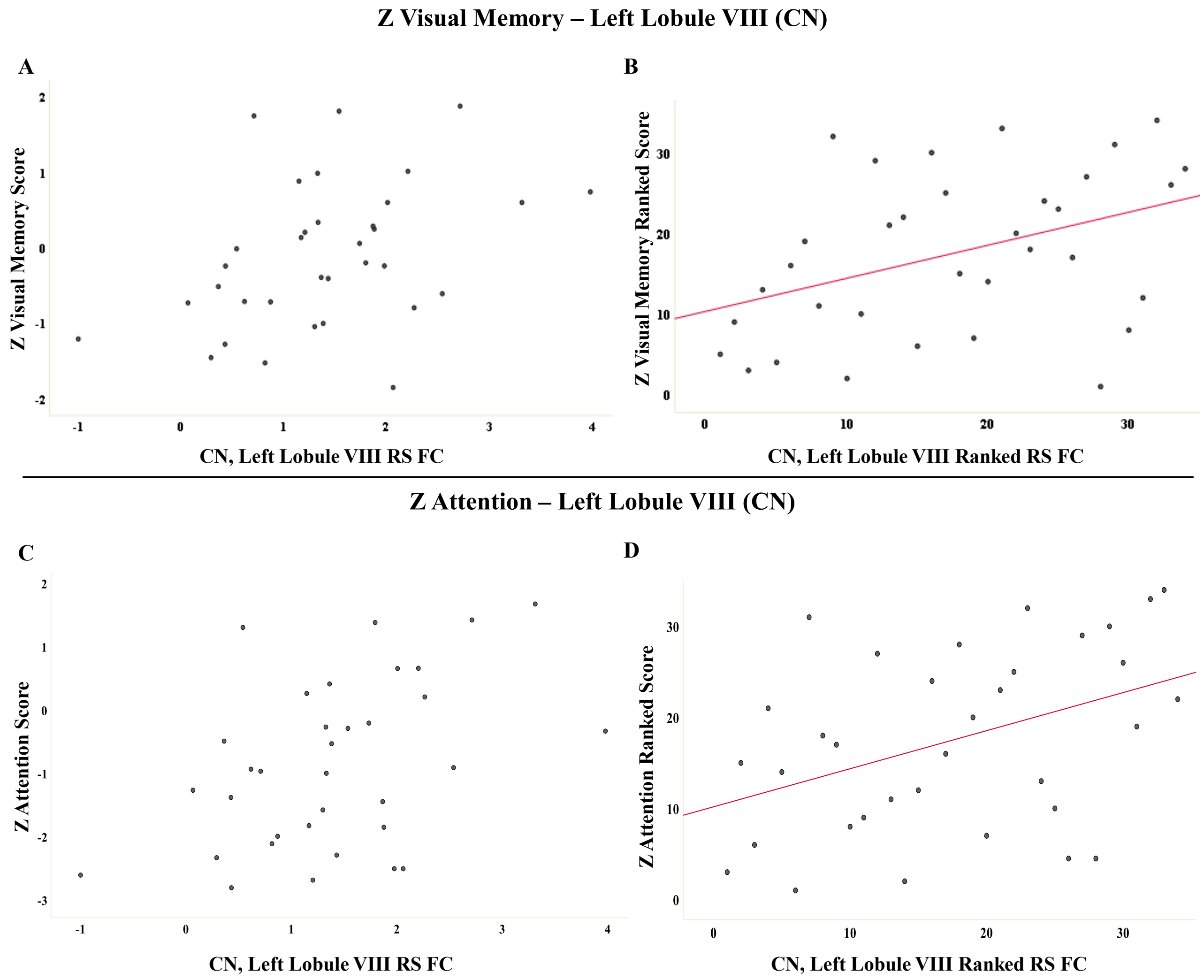

Supplementary Figure 5. Scatterplots illustrating the associations between cognitive domain z-scores and RS FC abnormalities within the CN in the LOMS cohort. Panels A and B show the association between visual memory and RS FC in the left lobule VIII, whereas Panels C and D show the association between attention and RS FC in the left lobule VIII. Panels A and C display actual observed values to show the underlying data distribution, whereas Panels B and D display rank-transformed data consistent with the Spearman correlation analyses. The red lines in Panels B and D represent the linear fit for visualization purposes only. Each datapoint represents one patient. In exploratory analyses, better visual memory ($\rho = 0.40$, 95% CI [0.05; 0.66], $p = 0.02$) and attention ($\rho = 0.42$, 95% CI [0.02; 0.70], $p = 0.03$) were associated with increased RS FC in the left lobule VIII. Sensitivity analysis excluding potentially influential observations identified using Cook's distance showed that all associations identified in the original analysis retained their statistical significance (Supplementary Table 4).

Abbreviations: CI=confidence interval; CN=cerebellar network; FC=functional connectivity; LOMS=late-onset multiple sclerosis; RS=resting state; Z=z-score.

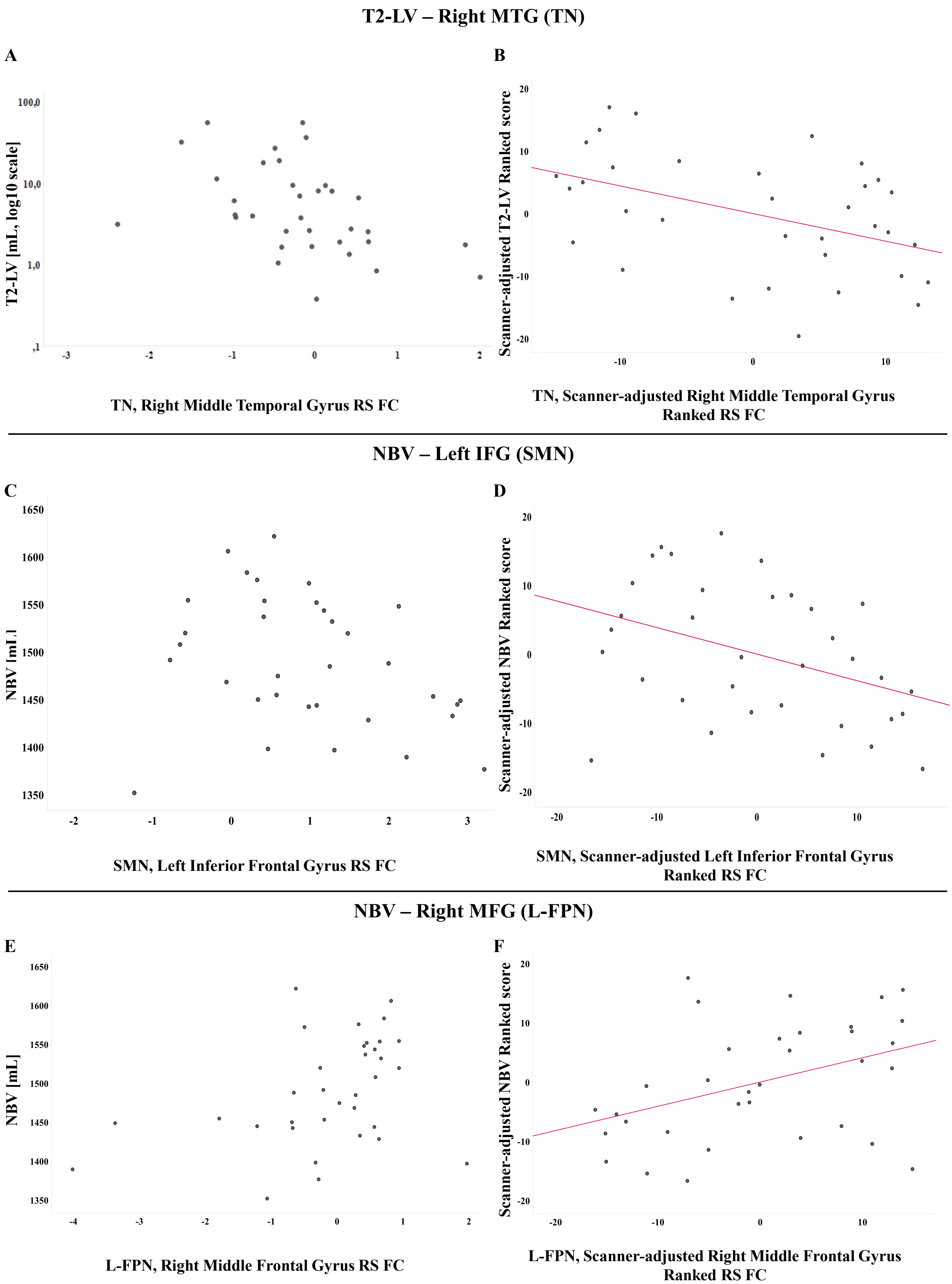

Supplementary Figure 6. Scatterplots illustrating the associations between MRI structural measures and RS FC abnormalities in the LOMS cohort. Panels A and B show the association between T2-LV and RS FC in the right MTG within the TN. Panels C and D show the association

between NBV and RS FC in the left IFG within the SMN, and Panels E and F show the association between NBV and RS FC in the right MFG within the L-FPN. Panels A, C, and E display actual observed values to show the underlying data distribution; because of its skewed distribution, T2-LV in Panel A is displayed in mL using a base-10 logarithmic axis for visualization purposes. Panels B, D, and F display scanner-adjusted rank-transformed data consistent with the partial Spearman correlation analyses described in the Methods. The red lines in Panels B, D, and F represent the linear fit for visualization purposes only. Each datapoint represents one patient. In exploratory analyses, higher T2-LV was associated with decreased RS FC in the right MTG within the TN ($\rho = -0.45$, 95% CI [−0.70; −0.11], $p = 0.01$), lower NBV was associated with increased RS FC in the left IFG within the SMN ($\rho = -0.39$, 95% CI [−0.65; −0.05], $p = 0.03$), and lower NBV was also associated with decreased RS FC in the right MFG within the L-FPN ($\rho = 0.41$, 95% CI [0.08; 0.67], $p = 0.02$). Sensitivity analysis excluding potentially influential observations identified using Cook's distance showed that all associations identified in the original analysis retained their statistical significance (Supplementary Table 4).

Abbreviations: IFG=inferior frontal gyrus; FC=functional connectivity; L-FPN=left fronto-parietal network; LOMS=late-onset multiple sclerosis; MFG=middle frontal gyrus; mL=milliliters; MTG=middle temporal gyrus; NBV=normalized brain volume; RS=resting state; SMN=sensorimotor network; T2-LV=T2 lesion volume; TN=thalamic network.
